# Assessment of Physico-Chemical Behavior and Sorptivity—Diatomaceous Earth as Support for Paraffinic Phase-Change Materials

**DOI:** 10.3390/ma17194691

**Published:** 2024-09-24

**Authors:** Agnieszka Przybek

**Affiliations:** Department of Materials Engineering, Faculty of Material Engineering and Physics, Cracow University of Technology, Jana Pawła II 37, 31-864 Cracow, Poland; agnieszka.przybek@pk.edu.pl

**Keywords:** diatomite, sorptivity of diatomaceous earth, medium for paraffin, properties of material

## Abstract

Diatomite’s most common application is its use as a sorbent for petroleum substances. Since paraffin is a petroleum derivative, this paper investigates the sorption capacity of diatomite to absorb it. In this paper, the physical and chemical properties were studied for 4 different fractions of diatomite (0–0.063 mm; 0–2 mm; 0.5–3 mm; and 2–5 mm) in the crude and calcined states, and the sorption capacity of diatomite earth for absorbing paraffinic phase-change substances was determined. The physical and chemical studies of the material included conducting an oxide chemical composition analysis using XRF, examining the composition of the mineral phases using X-ray diffraction, and determining the particle size, porosity, and thermal conductivity of the diatomite. Morphology images were also taken for all 8 diatomite variants using scanning electron microscopy. Each fraction was subjected to static calcination at 850 °C for 24 h. The results showed that the calcination of the diatomite increased the porosity of the material and reduced the thermal conductivity coefficient, and most importantly, the sorption capacity to absorb paraffins. The highest sorption capacity was characterized by calcined diatomite powder, that is, diatomite with the smallest particle size. Absorption of paraffinic substances by diatomite exceeding 200 wt.% is possible. Thus, diatomite is one of the feasible candidates for an economical and lightweight building material for making PCM composites for thermal energy storage in buildings.

## 1. Introduction

**Figure 1:**
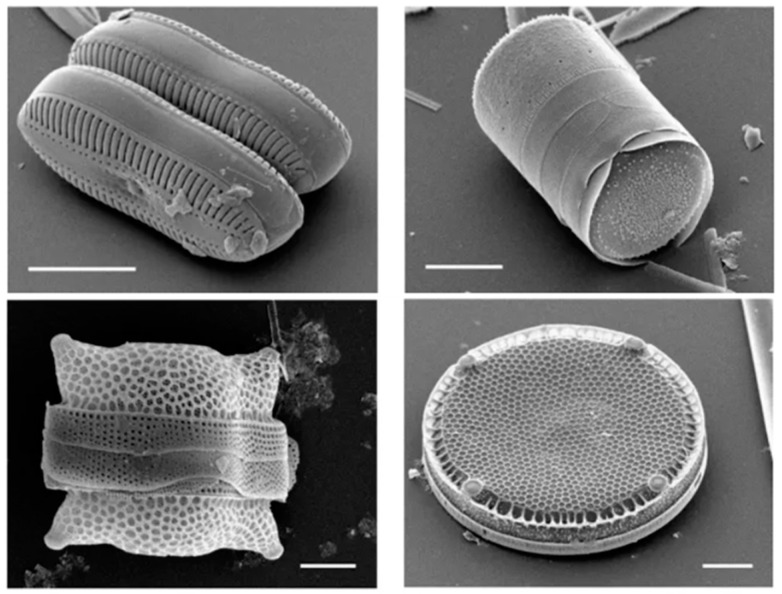
Diatom shells [11].

Diatomite, also known as diatomaceous earth, is a white or cream-colored, friable, porous rock. It is made up of the fossilized remains of diatoms, which are single-celled aquatic plants with cell walls of silica (a white or colorless crystalline compound) [1,2,3,4,5]. Diatoms are a type of algae that extract silica from water to build cell walls, and when they die, their silica-rich shells accumulate on the ocean floor or in freshwater bodies, eventually forming diatomite deposits [6,7,8,9,10]. Figure 1 presents the shells of diatoms [11].

Diatomite formation involves several stages [12,13,14]:

✓ Growth of diatoms: Diatoms, or microscopic algae, grow in aquatic environments such as oceans, lakes, and rivers.
✓ Silica extraction: Diatoms extract silica from water and use it to build complex and porous cell walls, called shells.
✓ Death and accumulation: When diatoms die, their shells sink to the bottom of the water body, accumulating over time. The accumulation of diatom material can result in the formation of layers or sediments.
✓ Consolidation: Over millions of years, accumulated diatomaceous material compacts and consolidates, resulting in diatomite rock.
✓ Mining: Diatomite deposits are usually mined in quarries or pits, and the material is processed to produce the desired product, which is often a fine powder.

Both the chemical composition and physical structure of diatomite make it suitable for many scientific and industrial purposes. Diatomite is a low-cost source of natural nanomaterials. Diatomite earth mainly contains amorphous silica and small amounts of impurities such as oxides of iron, aluminum, and calcium [15,16,17,18]. Diatomite is characterized by a highly porous microstructure (80–90% pores), low density, large surface area, high adsorption capacity, low thermal conductivity, and high melting point [19,20,21,22,23].

**Figure 2:**
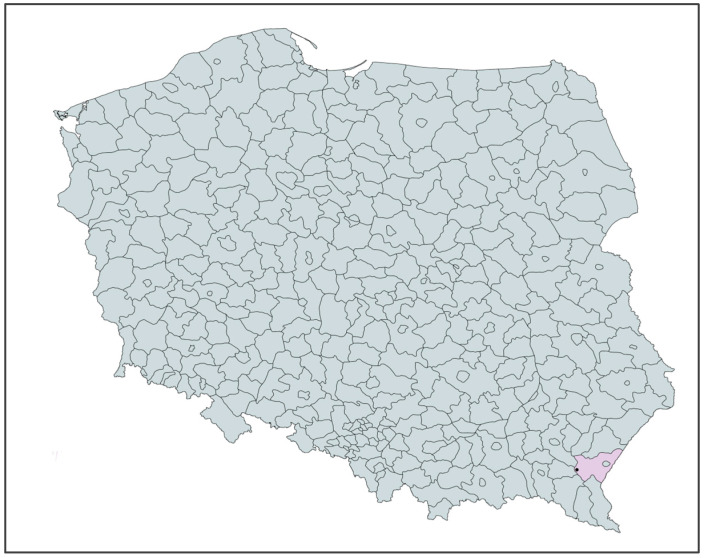
Map of Poland—location of diatomite mines.

In Poland, the only open-pit diatomite mine is located in Jawornik Ruski, in the Przemyśl district of Podkarpackie voivodeship. Figure 2 shows the location of the mine. The diatomite deposit was discovered in 1975, an experimental mining project was developed in 1976, and the mine was established in 1977 [24,25]. Since 1992, the diatomite deposit has been exploited by the only domestic producer of diatomite raw materials—the Specialized Mining Company GÓRTECH Ltd. [26]. The diatomite ore mined and processed at the mine site is processed mainly into sorbent, which is a means of removing oil spills [27,28,29]. Diatomite from Jawornik Ruski has an oil-derived substance absorption capacity of 100% by weight in the raw state [30].

Diatomite has a wide range of applications due to its unique properties [31,32]. Diatomite is widely used as a filtration aid. Its porous structure and large specific surface area make it effective in removing impurities from liquids, such as in producing beer, wine, and various industrial processes [33,34]. Diatomite’s high absorption capacity makes it useful in applications such as cat litter and spill absorbers [35]. Diatomite is used as a natural insecticide. It absorbs lipids from the waxy outer layer of insects, leading to dehydration and death [36,37]. Diatomite is also used in agriculture as a soil conditioner and additive to improve water retention and soil nutrient content [38,39,40]. It is also used in various industrial applications, including ceramics, paints, plastics, and construction materials [41,42]. Diatomite is also used in the food and beverage industry for filtration in the processing of sugar, oils, and beverages [43]. It is also used in some medical and cosmetic products as a mild abrasive and thickening agent [44,45]. Due to its remarkable properties, diatomite is used in various industries and has become an indispensable resource in a wide range of applications.

It is also possible to calcine diatomite, which further improves the sorption properties of this material. Researchers from the Cracow University of Technology proved in their work that the calcination of diatomite improved its absorbency by as much as 60 wt.% [30]. The most common application of diatomite is its use as a sorbent for petroleum substances. Since paraffin is a petroleum derivative, the present study examined the sorption capacity of diatomite to absorb it.

The impact of diatomite on sustainability, particularly in terms of the environmental consequences following mining or recycling, is multifaceted. Diatomite, a naturally occurring sedimentary rock composed primarily of silica from fossilized diatoms, has various applications that can either mitigate or exacerbate environmental issues depending on its use and the methods employed in its extraction and processing. Firstly, the mining of diatomite can lead to significant environmental disturbances. The extraction process typically involves surface mining, which can disrupt local ecosystems, lead to habitat destruction, and result in soil erosion. The heavy machinery used in mining operations can compact the soil and alter the landscape, potentially affecting water drainage patterns and local flora and fauna [46]. Moreover, the physical techniques employed in the beneficiation of diatomite, such as acid leaching and flotation, can generate substantial amounts of wastewater, which may contain harmful chemicals that pose risks to surrounding water bodies [47]. These environmental impacts underscore the need for sustainable mining practices that minimize ecological disruption and manage waste effectively. On the other hand, diatomite exhibits several beneficial properties that can contribute to environmental sustainability when used appropriately. Its high porosity and surface area make it an effective adsorbent for pollutants, including heavy metals and organic compounds, which can be particularly useful in water treatment applications [48,49]. For instance, diatomite has been shown to effectively filter heavy metals from contaminated water, thus playing a role in remediation efforts [48]. Additionally, its application in soil amendments can stabilize heavy metals, reducing their bioavailability and potential toxicity [50]. This dual role of diatomite as both a pollutant adsorbent and a stabilizing agent highlights its potential for a positive environmental impact when used in remediation strategies. Furthermore, the recycling of diatomite can enhance its sustainability profile. Recycled diatomite can be repurposed in various applications, including as a filler in construction materials and in the production of composite materials [51]. This not only reduces the demand for virgin diatomite but also minimizes waste, contributing to a circular economy. The incorporation of recycled diatomite into asphalt mixtures, for example, has been shown to improve its mechanical properties while reducing the environmental pollution associated with traditional asphalt production [52]. Such applications demonstrate how recycling diatomite can lead to more sustainable construction practices. In conclusion, while the mining of diatomite poses certain environmental challenges, its applications in pollution control and material recycling present significant opportunities for enhancing sustainability. The key to maximizing the environmental benefits of diatomite lies in adopting responsible mining practices and promoting its use in applications that contribute to ecological remediation and resource efficiency.

This manuscript investigated the sorption capacity of various fractions of raw and calcined diatomite to absorb paraffinic substances. Although research results confirm this property, the effect of thermal treatment and the degree of fineness of the diatomite on this characteristic have not been investigated so far. To date, no such studies have been conducted and no way has been identified to optimize the sorptivity of diatomite by controlling the degree of fineness and calcination of the material. The results of the conducted research indicate a significant contribution to the development of this field of science and may lead to a wider use of diatomites as materials to support paraffin absorption. This research is innovative due to the peculiarities of the diatomite material found in Poland and the fact that the absorption capacity of paraffinic phase-change materials will be different for diatomite found in other parts of the world. Since only the effect on the absorption of petroleum substances is known, it is possible to determine whether the degree of fragmentation and thermal treatment affect the absorption capacity of paraffin. Diatomite, which is known to be an environmentally friendly material, can therefore be used to support PCM stabilization. Taking all of the above factors into account, diatomite is one of the feasible candidates for an economical and lightweight building material for making PCM composites that will store thermal energy in buildings. Thanks to this property, the macroencapsulation of phase change materials in large quantities is possible. The results of the presented research are important not only for scientific development but also for the possibility of the commercialization of diatomite aggregates with paraffinic phase-change substances for insulation applications that improve the thermal comfort of buildings. The solution will result in a reduction in daily temperature amplitudes inside buildings and a phase shift in the time of the release of stored heat. It will be possible to use them not only for the exterior walls of buildings, but also for floors, windows and blinds, or even heat storage tanks.

## 2. Materials

### 2.1. Diatomite

**Figure 3:**
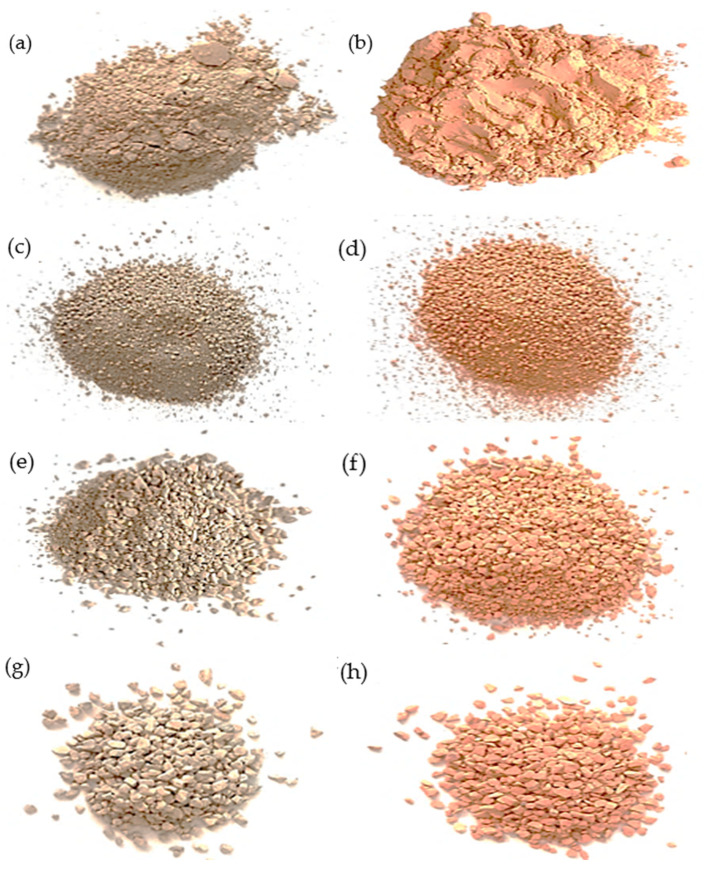
Diatomite: (**a**) diatomite powder, (**b**) diatomite powder after calcination, (**c**) granules, (**d**) granules after calcination, (**e**) 0.5–3 mm aggregate, (**f**) 0.5–3 mm aggregate after calcination, (**g**) 2–5 mm aggregate, and (**h**) 2–5 mm aggregate after calcination.

The material used for the study was diatomite. The diatomite came from the only mine in Poland located in Jawornik Ruski in the Podkarpackie Voivodeship (Specialized Mining Company Górtech Ltd., Cracow, Poland). In the present study, 4 different fractions of materials were used, differing in grain diameter: diatomite powder (0–0.063 mm), diatomite in the form of granules (0–2 mm), and two types of aggregates (0.5–3 mm and 2–5 mm). Each fraction was subjected to static calcination at 850 °C. Calcination was carried out in an FCF 7 SHM muffle laboratory furnace for 24 h. The calcination of diatomite was carried out at 850 °C, due to the best sorption capacity of this variant of the material, based on previous results of researchers from the Cracow University of Technology [30,53,54]. The temperature was selected to be sufficiently high to enable dehydration, dehydroxylation, and the removal of organic substances, while avoiding the decomposition of minerals and preventing the formation of liquid phases (sintering). Diatomite calcined at 850 °C demonstrates ideal properties for vibration damping, thanks to the specific structural and compositional changes that result from the calcination process. Thermal treatment at this temperature improves the mechanical strength and porosity of diatomite, which are key factors in its effectiveness for damping vibrations. Treatment at 850 °C significantly modifies the diatomite’s microstructure, increasing its specific surface area and porosity. This transformation is important because higher porosity enables better energy dissipation through the material under vibrational stress. The optimal pore structure formed at this temperature allows the material to efficiently absorb and dissipate vibrational energy, making it suitable for use in construction and material engineering. Additionally, the thermal treatment enhances the material’s thermal stability and reduces its density, further contributing to its vibration-damping capabilities. The balance between porosity and strength at this temperature is crucial for applications that require effective energy absorption and dissipation [55,56,57]. Diatomite before calcination is characterized by a gray/brown color, while after calcination it turns orange. Figure 3 shows the materials tested. A total of 8 different variants of diatomite were selected for the study to compare the physico-chemical properties of the material and its sorption capacity to absorb paraffinic substances.

For all 8 diatomite variants, oxide chemical composition analysis was carried out using the X-Ray Fluorescence (XRF) method. The tests were performed on a SCHIMADZU EDX-7200 (SHIMADZU Europa GmbH, Duisburg, Germany). All tests were carried out in an air atmosphere with Mylar films and holders designed for free-flowing materials, and the results are shown in Table 1. The table below shows only the most important oxides detected in the chemical composition, whose weight share is higher than 0.1. The oxide composition of all analyzed materials is similar. Each diatomite fraction contains the most SiO_2_ and Al_2_O_3_, with about 74–80 wt.% silicon dioxide and 12–15 wt.% diglinium trioxide. Other oxides that have been detected in the chemical composition of diatomite are Fe_2_O_3_, K_2_O, SO_3_, TiO_2_, and CaO.

**Figure 4:**
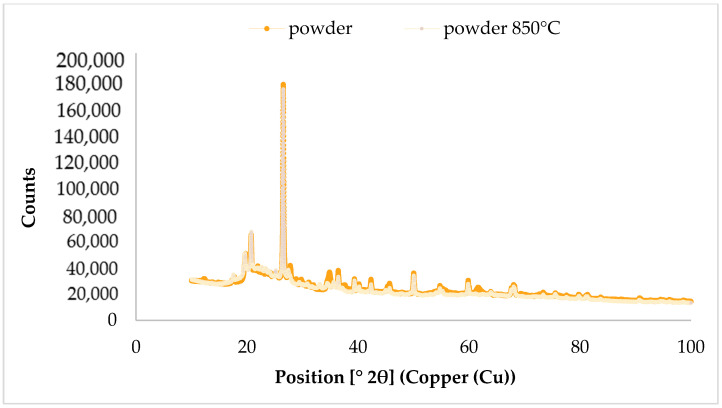
Patterns of X-ray diffraction for raw diatomite powder and calcined diatomite powder.

The composition of the mineral phases of the various diatomite fractions was studied by X-ray diffraction (XRD) technique, using a PANalytical Aeris diffractometer (Malvern Panalytical, Almelo, The Netherlands). Quantitative analysis was carried out using the Rietveld method, which was implemented in HighScore Plus software (version 4.8). Diffractograms were recorded using Cu-Kα radiation in a scan range of 10–100° with a step of 0.003° (2θ) and a time per step of 340 s, was used. X-ray diffraction patterns are shown in Figure 4 for only one selected fraction (in raw and calcined form), due to the fact that the diffractograms are quite similar. Meanwhile, the results of the quantitative analysis are summarized in Table 2.

For most of the diatomite variants analyzed, studies have shown that after calcination, a higher proportion by weight of kaolinite can be observed. Since the calcination process involves dehydration of the material at high temperatures, more metakaolin is formed in the diatomite, but this is an amorphous phase that cannot be detected by this test method. All detected phases are crystalline phases, so kaolinite was identified as a proxy for metakaolin. Mineral phases such as quartz, illite, kaolinite, and albite were identified for each diatomite variant.

### 2.2. Paraffinic Materials

Nine paraffinic substances were selected for diatomite absorption testing, including two typically petroleum-based substances for comparison (summer and winter crude). Winter fuel is a modified version of summer fuel. Gasoline has a higher vapor pressure and moisture-absorbing additives. Winter diesel has a lower water precipitation temperature and paraffin. Among the 9 variants of research reagents, there were also 3 commercially available paraffin-based phase-change materials. Two phase-change materials from Rubbitherm (Rubitherm Technologies GmbH, Berlin, Germany)—RT0 and RT25HC—and the PlusICE A18 phase-change material from PCM Products Ltd. (Peterborough, UK) were used. Polyethylene glycol 600 (Pol-Aura|Chemical Reagents, Belmont, NC, USA), light and heavy liquid paraffin from WARCHEM (Zakręt, Poland), and pharmaceutical-grade paraffin oil from FLEXOL (Ankleshwar, India) were also purchased. Each of the analyzed materials was in liquid form at room temperature. Table 3 shows the characteristics of paraffinic substances, based on data available from manufacturers [58,59,60,61,62].

## 3. Experimental Methods

### 3.1. Laser Particle Size Analysis for Diatomite

Using an Anton-Paar PSA 1190LD laser particle size analyzer (Anton-Paar, Graz, Austria), diatomite particle characterization tests were carried out. Particle size tests were carried out only for the finest of the fractions—0–0.063 mm—due to the possibility of damaging the machine, since measurements of up to 2 mm particle diameter can be made on it. For raw and calcined diatomite, five wet test measurements were made (water was the dispersing agent), and then the mean and standard deviation were calculated using Kalliope Professional software (version 2.22.1).

### 3.2. Porosity for Diatomite

Material porosity tests were carried out on a PoreMaster 33 mercury porosimeter (Anton-Paar, Graz, Austria) over a range of low pressures. The low pressure range is from 0.2 to 50 psi. The PoreMaster 33 mercury porosimeter is an instrument for measuring the size and volume of pores in materials (pore sizes range from 6.4 nanometers to 1.100 microns). Tests were conducted for the largest fractions of crude and calcined diatomite (between 0.5–3 mm and 2–5 mm) to get an idea of what the porosity distribution looks like.

### 3.3. Thermal Conductivity for Diatomite

Using the HFM 446 plate apparatus (Netzsch, Selb, Germany), thermal conductivity measurements were made for all diatomite variants. The plating apparatus operates based on the main standard EN 12664 [63] and other equivalents of these standards: ASTM C1784 [64], ASTM C518 [65], and ISO 8301 [66]. The accuracy of a single measurement is ±1–2%, and temperature regulation and control were verified by an advanced Peltier system. The thermal conductivity coefficient of diatomite was determined using the given device based on the cold and hot plate method. The lambda coefficient was tested in the temperature range of 0–20 °C.

### 3.4. Absorption Tests for Diatomite

**Figure 5:**
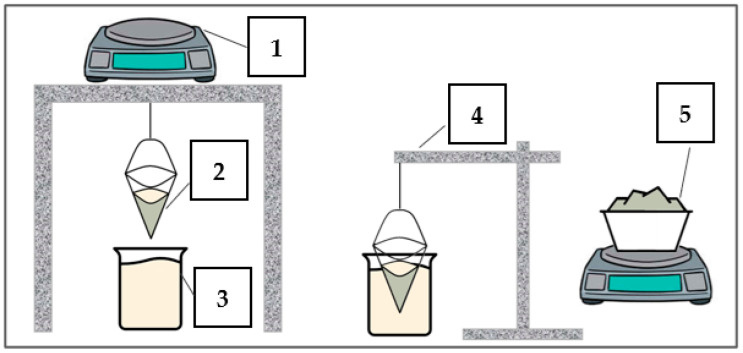
Diagram of test stand: (1) laboratory balance, (2) conical sieve, (3) test liquid, (4) tripod, and (5) test material.

Diatomite absorption tests were performed based on the Westinghouse method. For the study, 20 g of each material in the form of aggregates and 5 g of diatomite powder (due to its high volume) were used. The sorbent sample was placed on a sieve with a diameter of 100 mm and a height of 105 mm. Then, it was immersed in the test substance for 5 min and after that time it was translated to drain. On a special stand, the excess liquid was drained off for 10 min, and after a total time of 15 min, the sample was weighed. In the case of the powder, a mesh with a very small mesh size was additionally used to prevent the material from entering the test liquid. Each time, the sieve and the sieve with sorbent were weighed, as well as the sieve with the test substance and sorbent. The maximum absorbency of the sorbent was calculated as the ratio of the weight of the sorbent with substance to the sorbent without substance. All measurements were made using the comparative method. Figure 5 shows a schematic of the test stand.

### 3.5. SEM Morphology for Diatomite

Images of the morphology of the samples of all diatomite variants were taken using scanning electron microscopy (SEM). A JEOL IT 2000 microscope (JEOL, Akishima, Tokyo, Japan) was used for the study. To carry out the observation on the scanning electron microscope, each material was attached using a special EM-Tec C33 carbon adhesive and carbon disks. Both the adhesive and the disks were used to better attach the material and to lead to better conduction of the material. The samples were placed on metal tables and then in a holder, and immediately before testing, the surface of the material was coated with a conductive gold layer using a DII-29030SCTR Smart Coater vacuum sputtering machine (JEOL Ltd., Peabody, MA, USA).

## 4. Results

### 4.1. Laser Particle Size Analysis for Diatomite

Table 4 presents the particle size distribution for the raw and calcined diatomite powder. The calcination of the finest fraction of diatomite affected the increase in the diameter of powder particles. This phenomenon is probably due to the agglomeration of the smallest diatomite particles by partial sintering of the low-melting phases present in the material. This study was conducted using ultrasonication to eliminate the risk of agglomeration of the material into the dispersing agent—water. The observed increase in the diameter of diatomite particles after calcination at 850 °C is probably caused by the permanent bonding formed during the thermal treatment and the presence of a liquid phase.

### 4.2. Porosity for Diatomite

Table 5 summarizes the porosity tests for the largest diatomite fractions in the raw and calcined forms. The highest porosity and specific surface area were found in the 0–2 mm granules after calcination. The same granules obtained slightly lower results of these values before calcination. The largest fraction of diatomite both before and after calcination obtained a value of open porosity that was more than five times lower. The higher the average particle size of the material, the lower its specific surface area will be, as shown in the table below. Only inter-particle porosity was identified during the study; intraparticle porosity is not present in this type of material. Based on the following test results, it can be concluded that the smallest fractions of diatomite will have higher open porosity results, which will be proven in the next subsection. The porosity test results for the powder were performed using a different test method that is described in the authors’ paper [30].

### 4.3. Thermal Conductivity for Diatomite

For all fractions of diatomite, the thermal conductivity coefficient was tested before and after the thermal treatment. The results are presented in Table 6. The results show that the calcination process reduces the conductivity coefficient for each diatomite fraction. The lowest value of the heat conduction coefficient was recorded for the diatomite powder after calcination—0.070 W/m × K—while before calcination it was 0.092 W/m × K. The results for the other fractions are similar, settling at 0.152 W/m × K before calcination and 0.123 W/m × K after calcination.

### 4.4. Absorption Tests for Diatomite

**Figure 6:**
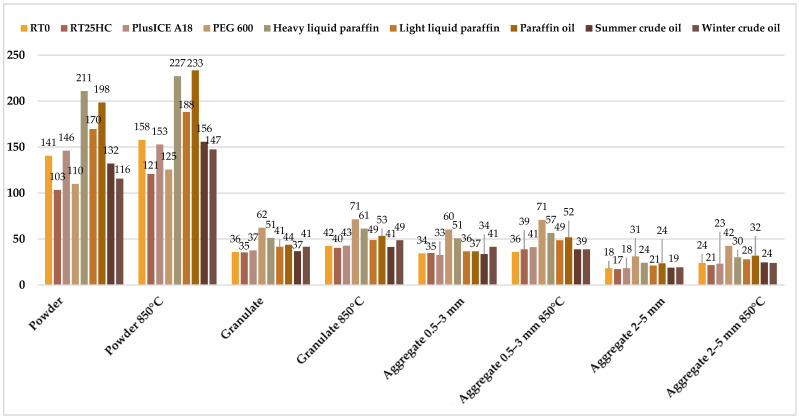
Absorption of paraffinic and petroleum substances by diatomite.

Figure 6 shows the results of the absorption of paraffinic phase-change substances and two petroleum substances by diatomite. The results show that absorption of paraffinic substances by diatomite exceeding 200 wt.% is possible. To compare the absorption capacity of paraffins and petroleum substances, the sorption capacity of summer and winter crude oil was also studied. Analyzing the chart below, calcined diatomite powder has the highest sorption capacity, which is the diatomite with the smallest particle size. For granules and aggregates, the absorption results are similar, but calcined diatomite shows a higher sorption capacity in each variant. The best absorption results were obtained for calcined diatomite powder for two substances: liquid heavy paraffin from WARCHEM and paraffin oil from FLEXOL. The absorption results for these two test liquids are 227 and 233 wt.%, respectively. As for the absorbency of petroleum substances, the best results settle at 150 wt.%. Given this, it can be concluded that diatomite, which was previously used as a sorbent for absorbing petroleum substances, absorbs paraffinic phase-change substances much better. The results of this study confirm that diatomite can therefore be an excellent medium for paraffinic phase-change substances, which can be used as a construction aggregate in lightweight geopolymer foams, the study of which will be presented in the authors’ next work.

### 4.5. SEM Morphology for Diatomite

**Figure 7:**
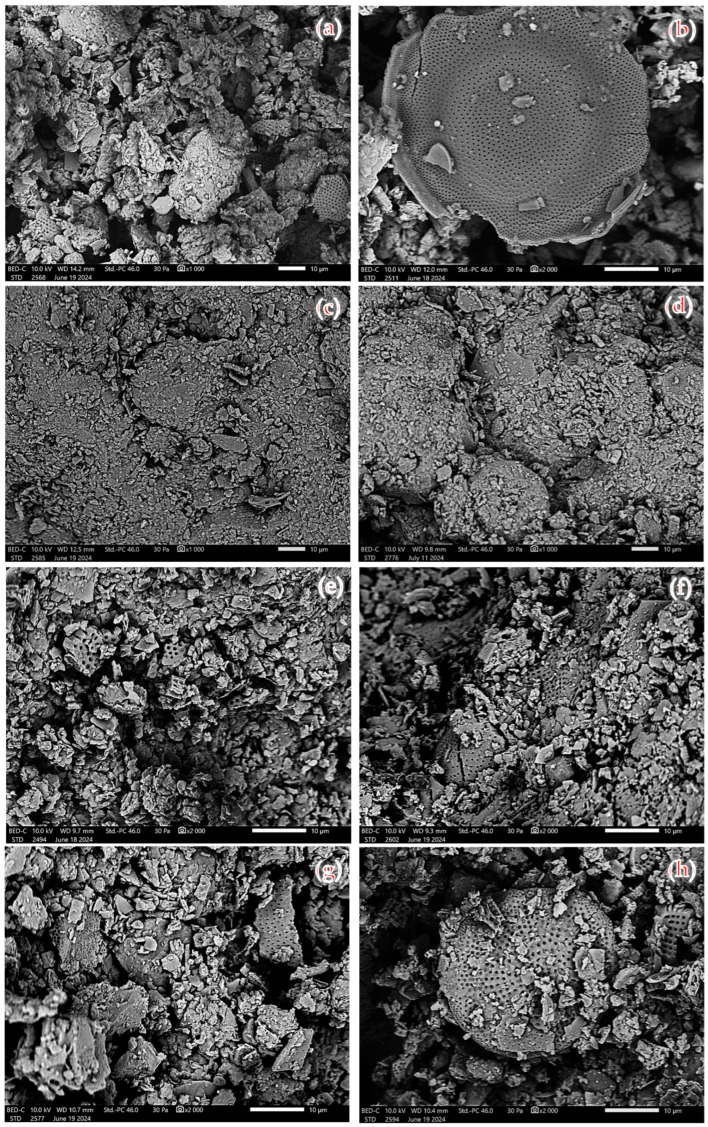
Morphology of diatomite: (**a**) diatomite powder, (**b**) diatomite powder after calcination, (**c**) granules, (**d**) granules after calcination, (**e**) 0.5–3 mm aggregate, (**f**) 0.5–3 mm aggregate after calcination, (**g**) 2–5 mm aggregate, and (**h**) 2–5 mm aggregate after calcination.

Figure 7 shows the results of the study of the morphology of the diatomite particles. All fractions of diatomite, which are described in this article, were studied. The following photographs mainly illustrate the different forms of diatomite and diatomite carapaces. The SEM photos illustrate the mechanically degraded diatoms, as well as the mainly disk-shaped diatoms. The morphology images show the different shapes and sizes of the diatoms, as well as the very high porosity of the diatomite structure. This high porosity is particularly important in terms of their ability to absorb test substances—in this case, paraffins. The microstructure of diatomite plays a very important role when it comes to the sorption capacity of this material. On the other hand, the sphericity of diatomite particles in granular form can be observed in Figure 7a,b. The SEM images were taken at a very high magnification of 1000 and 2000× to illustrate the form of the diatoms.

## 5. Discussion

In the present study, the physico-chemical properties were investigated for four different fractions of diatomite in the raw and calcined state, and the sorption capacity of the diatomite to absorb paraffinic phase-change substances was determined. Physical and chemical studies of the material included conducting oxide chemical composition analysis using XRF, examining the composition of mineral phases using X-ray diffraction, and determining the particle size, porosity, and thermal conductivity of the diatomite. Morphology images were also taken for all eight diatomite variants using scanning electron microscopy.

The analysis of the oxide chemical composition of the diatomite showed that the composition is similar regardless of the size of the material fractions. Each diatomite fraction contains the most SiO_2_ and Al_2_O_3_, with about 74–80 wt.% silicon dioxide and 12–15 wt.% diglinium trioxide. Other oxides that have been detected in the chemical composition of the diatomite are Fe_2_O_3_, K_2_O, SO_3_, TiO_2,_ and CaO. The analysis of the mineral composition of the diatomite, on the other hand, showed that after calcination, a higher weight share of kaolinite could be observed. This is because the calcination process involves dehydration of the material at high temperatures so that more metakaolin is formed in the diatomite. The analysis of crystalline phases was carried out using kaolinite, which was identified as a substitute for metakaolin, since metakaolin is an amorphous phase. Mineral phases such as quartz, illite, kaolinite, and albite were identified for each diatomite variant. Studies of the chemical and phase compositions of diatomite from Polish deposits conducted by other scientists are consistent with the results presented in this manuscript [28,30,31,67,68,69].

A particle size analysis was carried out for the diatomite powder, which showed that the powder in its raw form had an average particle size of 11.305 μm, while after calcination the material had a particle size of 14.691 μm. An increase in the particle diameter of diatomite after calcination at 850 °C was observed, and this is most likely caused by the permanent bonds formed during the thermal treatment and the presence of the liquid phase. Measurements of the average size of diatomite powder from Poland made by other researchers are very similar to those presented in this research work [30,53,54].

The porosity tests for the largest fractions of diatomite in the raw and calcined forms showed that the material with the smallest particle size distribution, i.e., 0–2 mm granules, had the highest porosity and specific surface area. The calcination process increases porosity. The largest fraction of diatomite both before and after calcination obtained a value of open porosity that was more than five times lower. The higher the average particle size of the material, the lower its specific surface area will be [70,71,72]. Only inter-particle porosity was identified during the study; intraparticle porosity is not present in this type of material. Based on the following test results, it can be concluded that the smallest fractions of diatomite (0–2 mm) have the highest porosity, which means they have the highest sorption capacity among the tested variants (0–2 mm, 0.5–3 mm, and 2–5 mm). The high porosity affects the high absorbency of the material [73,74,75].

For all diatomite fractions, the thermal conductivity coefficient before and after thermal treatment was studied. The results show that the calcination process reduces the conductivity coefficient for each diatomite fraction. The lowest value of the thermal conductivity coefficient was recorded for the diatomite powder after calcination—0.069 W/m × K—while before calcination it was 0.092 W/m × K. The results for the other fractions are similar, settling at 0.151 W/m × K before calcination and 0.122 W/m × K after calcination. The thermal conductivity of diatomite is in the range of 0.05–0.10 W/m × K, which coincides with the results presented [13].

The results of the study of the sorption capacity of diatomite earth showed that it is possible for the absorption of paraffinic substances by diatomite to exceed 200 wt.%. To compare the absorption capacity of paraffins and petroleum substances, the sorption capacity of summer and winter oil was also tested. Calcined diatomite powder, which is the diatomite with the smallest particle size, had the highest sorption capacity. For the granules and aggregates, the absorption results are similar, but the calcined diatomite showed higher sorption capacities in each variant [30]. The best absorption results were obtained for calcined diatomite powder for two substances: liquid heavy paraffin by WARCHEM and paraffin oil by FLEXOL. The absorption results for these two test liquids are 227 and 233 wt.%, respectively. As for the absorbency of petroleum substances, the best results settle at 150 wt.% and are similar to the results of other researchers [30]. Given this, it can be concluded that diatomite, which was previously used as a sorbent for absorbing petroleum substances, absorbs paraffinic phase-change substances much better. The results of this study confirm that diatomite can therefore be a great medium for paraffins. The sorption capacity of diatomite for absorbing paraffins is a topic that is still unexplored and innovative. The results of other researchers, however, have shown that they have obtained inferior absorption results [76]. Diatomite saturated with paraffinic phase-change substances may also have engineering applications in a low-scale refrigeration system and for electronic cooling [77,78].

## 6. Conclusions

Various fractions of diatomite in raw and calcined forms from the open-pit mine in Jawornik Ruski were tested. The results showed that the calcination of diatomite increases the porosity of the material and lowers the coefficient of thermal conductivity, and, above all, improves the sorption capacity to absorb paraffins. The highest sorption capacity was characterized in calcined diatomite powder, that is, the diatomite with the smallest particle size. Absorption of paraffinic substances by diatomite exceeding 200 wt.% is possible. The best absorption results were obtained for calcined diatomite powder for two substances: liquid heavy paraffin from WARCHEM and paraffin oil from FLEXOL. The dominant factor affecting the ability of diatomite to absorb paraffins is calcination, which is based on industrial practice and scientific research. The process of calcination is a significant factor in determining the ability of diatomite to absorb paraffin due to its profound effects on the material’s structural and chemical properties. Calcination, which involves heating diatomite to high temperatures, leads to significant changes in its surface area, porosity, and chemical composition, all of which are critical for enhancing absorption capacity. One of the primary outcomes of calcination is an increase in the surface area and porosity of diatomite. Calcination is an effective method for enriching diatomite, resulting in a higher surface area that is essential for adsorption processes. The increased porosity allows for a greater accommodation of paraffin molecules, facilitating their absorption. Furthermore, thermal treatment alters the microstructure of diatomite, creating a more favorable environment for paraffin absorption by enhancing the accessibility of the pores. This structural modification is crucial, as it directly correlates with the material’s ability to interact with and retain paraffins. In addition to physical changes, calcination also influences the chemical properties of diatomite. The process can lead to the formation of reactive sites on the diatomite surface, which can enhance its interaction with paraffins. The presence of reactive silica and alumina phases, generated during calcination, can significantly improve the adsorption capacity of diatomite by providing additional binding sites for paraffin molecules. This chemical modification is essential for enhancing the overall efficiency of diatomite as an absorbent material. All of the above factors confirm that diatomite is one of the feasible candidates for use as an economical and lightweight building material for PCM composites that will store thermal energy in buildings. Thanks to this property, the macroencapsulation of phase change materials in large quantities is possible. The results of the presented research are important not only for scientific development but also for the possibility of the commercialization of diatomite aggregates with paraffinic phase-change substances for insulation applications to improve the thermal comfort of buildings.

The author’s subsequent work will present the results of studies of diatomite granules capable of absorbing energy. Among other things, tests on the heat capacity and determination of the latent heat of fusion, as well as the thermal expansion coefficient of the finished material will be carried out. The main objective of this work was to determine the sorption capacity of diatomite to absorb paraffins. Organic phase-change materials were used in this study. The choice of this type of phase-change material was made for economic reasons. The use of inorganic phase-change materials would be a very interesting solution, but the overriding aim of this work is to produce an economical candidate for engineering applications. Phase-change materials are very expensive, especially inorganic phase-change materials, so this thesis only presents test results for substances that are commercially available and, in terms of financial aspects, cheap. The use of phase-change materials in construction has not been a common solution so far due to the high price of these materials, but this thesis aims to present a solution that is cheap and feasible.

## Figures and Tables

**Table 1:** Oxide analysis for different raw and calcined diatomite fractions.

Type of Diatomite	Oxide Composition (wt.%)						
	SiO_2_	Al_2_O_3_	Fe_2_O_3_	K_2_O	SO_3_	TiO_2_	CaO
Powder	78.697	15.991	2.752	1.427	0.447	0.367	0.235
Powder 850 °C	80.331	15.286	2.318	1.264	0.174	0.329	0.220
Granulate	74.117	14.849	6.120	2.480	0.930	0.610	0.610
Granulate 850 °C	74.920	15.320	5.660	2.370	0.350	0.580	0.560
Aggregate 0.5–3 mm	78.244	14.275	2.328	1.348	2.515	0.350	0.863
Aggregate 0.5–3 mm 850 °C	78.459	12.487	4.050	2.030	1.390	0.530	0.930
Aggregate 2–5 mm	79.420	15.558	2.090	1.416	0.624	0.365	0.447
Aggregate 2–5 mm 850 °C	77.048	14.057	4.680	2.160	0.750	0.540	0.630

**Table 2:** Mineral phase quantitative analysis for different raw and calcined diatomite fractions.

Type of Diatomite	Quantitative Share of Phase [%]			
	Quartz	Illite	Kaolinite	Albite
	01-075-8321	00-026-0911	00-058-2001	00-001-0739
	SiO_2_	Al_2_Si_3_AlO_10_(OH)_2_	Al_2_Si_2_O_5_(OH)_4_	NaAlSi_3_O_8_
Powder	19.0	23.7	39.9	17.4
Powder 850 °C	21.9	13.6	48.2	16.3
Granulate	26.3	28.5	28.4	16.8
Granulate 850 °C	22.2	12.2	47.6	17.9
Aggregate 0.5–3 mm	15.3	20.0	46.7	18.1
Aggregate 0.5–3 mm 850 °C	30.2	27.2	27.4	15.2
Aggregate 2–5 mm	17.3	24.2	40.2	18.3
Aggregate 2–5 mm 850 °C	26.9	24.0	22.2	27.0

**Table 3:** Characteristics of paraffinic substances.

Material	Phase Change Area [°C]	Thermal Conductivity [W/m × K]	Specific Heat Capacity [kJ/kg × K]	Flash Point [°C]	Density [g/cm^3^]	Viscosity [mPa × s]
RT0	−1 to 2	0.2	2	110	0.770–0.880	-
RT25HC	22 to 26	0.2	2	150	0.770–0.880	-
PusICE A18	18	0.22	2.18	200	0.765	-
PEG 600	17 to 22	0.187	-	252	1.127	16–19
Light liquid paraffin	−20 to −15	0.1–0.3	-	-	0.810–0.875	25–80
Heavy liquid paraffin	−12 to −10	0.1–0.3	-	-	0.845–0.880	110–230
Paraffin oil	−30 to −3	0.1–0.3	-	260	0.862	-

**Table 4:** Particle size distribution for raw diatomite powder and calcined diatomite powder.

Material	D_10_ [μm]	D_50_ [μm]	D_90_ [μm]	Average Particle Size [μm]	Standard Deviation [μm]
Powder	2.641	9.850	20.205	11.305	0.196
Powder 850 °C	3.974	13.708	24.371	14.691	0.127

**Table 5:** Porosity for largest fractions of diatomite (raw and calcined).

Material	Total Porosity [%]	Pore Diameter Range [μm]	Total Surface Area [m^2^/g]	Total Intruded Volume [cm^3^/g]
Granulate	52.61	4.26–1068.83	0.0088	0.3175
Granulate 850 °C	55.92	4.26–1068.83	0.0086	0.3321
Aggregate 0.5–3 mm	32.40	4.27–1051.31	0.0089	0.2810
Aggregate 0.5–3 mm 850 °C	35.83	4.25–1073.31	0.0104	0.2331
Aggregate 2–5 mm	11.67	4.30–906.43	0.0054	0.1498
Aggregate 2–5 mm 850 °C	9.67	4.26–1100.94	0.0034	0.0602

**Table 6:** Thermal conductivity for different raw and calcined diatomite fractions.

Type of Diatomite	Thermal Conductivity Coefficient λ [W/m × K]
Powder	0.092
Powder 850 °C	0.070
Granulate	0.152
Granulate 850 °C	0.137
Aggregate 0.5–3 mm	0.152
Aggregate 0.5–3 mm 850 °C	0.121
Aggregate 2–5 mm	0.143
Aggregate 2–5 mm 850 °C	0.123

## Data Availability

The original contributions presented in the study are included in the article, further inquiries can be directed to the corresponding author.
